# Pharmacological modulation of beta-catenin and its applications in cancer therapy

**DOI:** 10.1111/jcmm.12033

**Published:** 2013-03-14

**Authors:** Ravi Thakur, Durga Prasad Mishra

**Affiliations:** a Cell Death Research Laboratory, Division of Endocrinology, Central Drug Research InstituteLucknow, India

**Keywords:** β-catenin, Pharmacological inhibitors, Phytochemicals, Cancer

## Abstract

Beta-catenin (β-catenin) is a multifunction protein with a central role in physiological homeostasis. Its abnormal expression leads to various diseases including cancer. In normal physiology, β-catenin either maintains integrity of epithelial tissues or controls transcription of various genes on extracellular instigations. In epithelial tissues, β-catenin functions as a component of the cadherin protein complex and regulates epithelial cell growth and intracellular adhesion. In Wnt signalling, β-catenin is a major transcriptional modulator and plays a crucial role in embryogenesis, stem cell renewal and organ regeneration. Aberrant expression of β-catenin can induce malignant pathways in normal cells and its abnormal activity is also exploited by existing malignant programmes. It acts as an oncogene and modulates transcription of genes to drive cancer initiation, progression, survival and relapse. Abnormal expression and function of β-catenin in cancer makes it a putative drug target. In the past decade, various attempts have been made to identify and characterize various pharmacological inhibitors of β-catenin. Many of these inhibitors are currently being investigated for their anticancer activities in a variety of cancers. The first half of this review will focus on the role of β-catenin in cancer initiation, maintenance, progression and relapse whereas the second half will briefly summarize the recent progress in development of agents for the pharmacological modulation of β-catenin activity in cancer therapeutics.

- Introduction
- Role of β-catenin in cancer
- β-catenin inhibitors
- Plant-derived β-catenin modulators
- Conclusion and prospective

## Introduction

Beta-catenin (β-catenin) is the mammalian homologue of the drosophila armadillo gene 1. It acts both as a transcriptional co-regulator and an adaptor protein for intracellular adhesion 1–3. β-catenin is essential for the establishment and maintenance of epithelial layers and provides a mechanical linkage between intracellular junctions and cytoskeletal proteins 4, 5. Wnt signalling is the chief regulator of β-catenin 6, 7. Binding of the Wnt ligand to frizzled receptors hyper-phosphorylates and thus activates the dishevelled protein (dsh) 8. Hyper-phosphorylation of dsh results in the displacement of GSK-3β from the β-catenin degradation complex and prevents GSK-3β-mediated phosphorylation of β-catenin 8. This complex comprises adenomatous polyposis coli (APC), axin and GSK-3β 8. In the absence of Wnt signal, GSK-3β and casein kinase 1 (CK1) phosphorylate β-catenin 8, 9. Phosphorylation of β-catenin leads to its ubiquitination and proteasomal degradation through the F-box/WD repeat-containing protein 1A (FBXW1A)/S-phase kinase-associated protein (SKP) complex 8. When not degraded *via* the proteolytic pathway, β-catenin accumulates in the perinuclear region and forms a cytoplasmic pool of free signalling molecules 8, 9. Here, the stable β-catenin interacts with the lymphoid enhancer factor/T cell factor (LEF/TCF) and is translocated into the nucleus as a complex of β-catenin/LEF/TCF to stimulate target gene transcription by displacement of groucho-HDAC co-repressors 8, 10.

Various extracellular signals regulate the localization of β-catenin either on the membrane or in the cytoplasm 11. Activation of receptor tyrosine kinases (RTKs) or cytoplasmic tyrosine kinases (Fer, Fyn, Yes and Src), phosphorylate β-catenin at specific tyrosine residues Y654 and Y142 12. Y654 phosphorylation leads to the inhibition of the catenin/E-cadherin interaction, leading to the dissociation of the complex and subsequent degradation of E-cadherin and β-catenin 13. Dissociation of the E-cadherin–β-catenin complex leads to the loss of epithelial apico-basal polarity 14. At the same time presence of other signals decide cellular response to this change 11. Extracellular signals mediated through growth factors such as platelet-derived growth factor (PDGF), epidermal growth factor (EGF), insulin and insulin-like growth factor I (IGF-I) activate PI3K-AKT-MAPK or PKC pathways 15–19. Activation of these pathways promotes nuclear accumulation of β-catenin by inhibition of GSK3β and supports epithelial to mesenchymal transition (EMT). 15–19. These pathways also play a critical role in transforming epithelial tumours into an invasive forms and help in the progression of various fibrotic diseases 19. β-catenin accumulation within the nucleus or cytoplasm has been found in more than half of all cancer cases and is related to increased tumourigenicity 20–25. Cytoplasmic β-catenin is a hallmark of colon cancer 1. It can induce tumourigenic traits in normal cells, and further supports cancer cell proliferation and survival 19, 24. High-level cytoplasmic expression, and nuclear localization of beta-catenin, is characteristic of stem-like cell populations in cancers that are resistant to chemotherapeutics and capable of initiating new tumours 29, 30. β-catenin also helps in creating a suitable niche for cancer progression by modulating cancer microenvironment 18, 26–30.Various studies have shown that inhibition of β-catenin activity leads to suppression of several cancer hallmarks and is thus perceived as a putative drug target 31.

## Role of β-catenin in cancer

Accumulating evidence indicates that β-catenin has a central role in the malignant transformation of normal cells 32–36. Herencia *et al*. while studying hepatocyte differentiation in mesenchymal stem cells have found that the activation of Wnt signalling and β-catenin nuclear localization results in a tumoural phenotype 32. They reported an increase in the expression of liver cancer-related proteins in cells with high β-catenin nuclear localization 32. Heiser *et al*. observed rapid formation of lipogenic liver tumours in mice on AKT and β-catenin co-activation 33. In pancreatic cells and lung epithelial cells, activation of β-catenin has also been reported to be sufficient for induction of oncogenic transformation 34, 35. A recent study demonstrated that Wnt/β-catenin pathway activation in cerebellar progenitor cell prevents terminal differentiation of these cells and maintain them in a stem cell like state 36. This study further suggested that medulloblastoma can also originate from cells other than granule progenitors. Wnt/β-catenin pathway is also a major regulator of cancer initiating cell (CIC) genesis 36. Oncogenic mutants of β-catenin have also been reported and the prevention of their degradation results in intracellular accumulation. These mutants can induce tumour formation in transgenic animals 37, 38. The importance of β-catenin in abnormal cell proliferation attained prominence after the discovery of β-catenin oncogenic mutations in APC wild-type colon cancers 3, 39. Mutant β-catenin protein is not degraded by APC, thus leading to its accumulation in the cytoplasm resulting in uncontrolled cellular proliferation 39. The frequency of oncogenic mutations in β-catenin is low but has been reported in a variety of human cancers 40. Several studies have shown (reviewed elsewhere) that β-catenin is a key modulator of cancer cell proliferation and survival 4, 41. Initial key studies carried out by Tetsu *et al*. and Shtutman *et al*. in colon cancers revealed that β-catenin activates transcription from the cyclin D1 (CCND1) promoter, and consensus TCF/LEF-binding sites are necessary for this activity 42, 43. β-catenin/TCF/LEF transcription activity also regulates expression of c-Myc, TP63 isoform ΔN (ΔNp63), microphthalmia-associated transcription factor (MITF), limb bud and heart development homolog (LBH), survivin and c-Jun in various cancer models 44–49. c-MYC and c-JUN act as oncogenes in their active state, while ΔNp63, CCND1, MITF, LBH perform various functions to support cell growth and survival 44–48. A list of β-catenin target genes in various cancers is briefly summarized in Table 1. β-catenin has also been found to support tumour growth by promoting angiogenesis in cancers 49. It regulates expression of vascular endothelial growth factor (VEGF) 49. Collectively, these studies indicate that β-catenin has an important role in maintaining malignancies by supporting cell proliferation and survival.

**Table 1:** Major β-catenin target genes in cancer

S.NO.	Gene Name	Function
1	MYCBP 113, JAG1 114, MSL1 11, PPARdelta 110,	Cell transformation
2	CCND1 42, 43, c-myc 44, ΔNp63 45, MITF 46, LBH 47, survivin 48 and c-Jun 49, fra-1(Fosl1) 49, FGF18 107, Hath1 [108], MET 109, FGF9 112	Cell growth, Proliferation and survival
3	MMP2 51, MMP9 51, MMP-7 52, MMP26 54, VEGF 48, TIAM1^112^, TWIST1 115, SNAI2 57, ZEB1^116^, S100A4 58, uPAR 49	Migration, Invasion, EMT
4	CD44 104, VEGF 48, BMP4 106, Ephb 105, GREM1 110, EDN1^103^	Progression, Angiogenesis and Niche establishment
5	CD44 104, HTERT 117, NANOG 117, OCT4 118	Cancer stem cells

Metastasis is an important cancer hallmark and it is often supported by abnormal β-catenin expression or localization 50. β-catenin supports the metastatic programme by increasing the migratory and invasive capabilities of cancer cells 18, 42. It regulates expression of various invasion-related genes like matrix metalloproteinases (MMP2, MMP7, MMP9, MMP26) 51–54. β-catenin also regulates EMT, which can endow cells with higher invasive, metastatic and survival potential 26. EMT-like state in cancers is promoted by activation of Snail1 (Snail), Snail2 (Slug), ZEB1, CBF-A/KAP-1 complex, Twist, LEF-1, Ets-1, FOXC2 and Goosecoid transcription factors (TFs) 26. These TFs work downstream of various growth factor (EGF, TGF-β and IGF1) signalling pathways initiated by changes in cancer microenvironment 55. Snail and slug transcription factors help in the formation of β-catenin/LEF-1 transcription complex and promote expression of transforming growth factor 3-beta (TGFβ3) to induce EMT 56. β-catenin/LEF1 also regulates expression of Snail, LEF1 and other EMT markers at the transcriptional level 57. β-catenin regulates expression of metastasis-associated gene S100A4 and Tenascin C (TNC) 58, 59. TNC is an extracellular matrix (ECM) protein 60. It supports the growth and proliferation of metastasis-initiating cancer cells and acts as an important ECM component of the metastatic niche 60. Nuclear localization of β-catenin has been postulated as a potential marker for local lymph node or distant metastasis in variety of cancers including oesophageal, breast, colorectal, prostate, lung and cervical cancer 61–65. Apart from the increased expression or nuclear localization a decrease in the β-catenin expression has been observed in melanoma, prostate, thyroid and gastric cancers 66–69. Decreased β-catenin level in these cancers was associated with their increased metastatic potential 66–69. This probably indicates that breakdown of normal β-catenin functions can also govern cancer progression and requires further investigation. Cancer cells are associated with various normal stromal cells called cancer-associated cells 70. Fibroblasts, macrophages, regulatory T cells, mesenchymal stem cells (MSCs) and endothelial cells are the common members of the cancer stroma 70. These cells in the cancer microenvironment support cancer growth and progression. In oesophageal cancers, tumour-associated fibroblasts are responsible for higher expression and nuclear localization of β-catenin in adjacent cancer cells 71. A recent study indicated that nuclear overexpression of β-catenin in tumour-associated fibroblasts is a good prognostic indicator in breast cancers 72. The study also reported that the ectopic expression of β-catenin in fibroblast increases proliferation and invasion of co-cultured cancer cells 72. Fibroblasts in co-culture have also been shown to increase expression of β-catenin in breast cancer cells 73. It also increases proliferation of CD44+/CD24low/- (CSC) subpopulation to a fivefold higher level than that of the normal breast cancer cells 73. These studies underscore the importance of β-catenin in regulating tumour microenvironment. However, the low β-catenin expression associated with metastasis needs further investigation. Collectively, β-catenin activity is modulated by extracellular changes and in response it modulates cancer microenvironment to promote tumour growth, invasion and metastasis 18, 55.

**Fig. 1:**
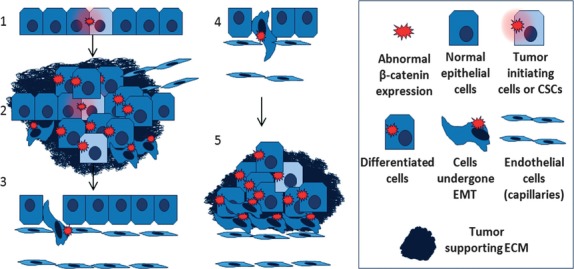
Role of β-catenin in Tumourigenesis. Beta-catenin supports: (**A**) transformation of normal cells to cancerous one. (**B**) Cancer cell proliferation, renewal, differentiation, niche establishment, angiogenesis and EMT. (**C**) Invasion and Intravasion. (**D**) Extravasion. (**E**) tissue invasion and organ homing to establish micrometastasis. CSC, Cancer stem cell; EMT, Epithelial to mesenchymal transition; ECM, Extracellular matrix.

Abnormal activity of β-catenin is further associated with cancer drug resistance and cancer stem cell state 29, 30. It thus associated with poor patient outcome and disease relapse 29, 30, 74–77. β-catenin is essential for the self-renewal of normal as well as cancer stem cells. Zhao *et al*. explored the role of β-catenin in haematopoietic malignancies 74. They found that β-catenin plays an essential role in AML and CML development and also helps in cancer stem cell renewal 74. Various other studies have postulated that angiogenesis, presence of highly resistant cancer stem cells (CSCs), EMT, deregulation of cell cycle and apoptosis are central wheels in mechanisms of cancer aggressiveness and chemoresistance 78, 79. Current knowledgebase suggests that Wnt/β-catenin signalling has a role in all these five aspects associated with the process of carcinogenesis. It plays an essential role in cancer initiation, maintenance, progression, survival and relapse 18, 26, 32, 57, 74–76. Owing to its place at the heart of malignant programmes, β-catenin is increasingly perceived as a putative drug target (Fig. 1).

## Beta-catenin inhibitors

Inhibition of β-catenin using small molecule inhibitors or siRNA abrogates tumour growth 80, 81. In the year 2002, Kim *et al*. for the first time demonstrated that specific inhibition of the oncogenic form of β-catenin is sufficient to reverse the transformed properties of human cancer cells 82. In their study, they found that β-catenin is a necessary oncogene and the pharmacological inhibition of oncogenic β-catenin is likely to be an effective strategy for reversing the malignant properties of advanced human tumours 82. To date, many β-catenin signalling pathway inhibitors are under investigation with the potential aim of disrupting β-catenin activity and its interaction with the transcription factors. Lepourcelet *et al*. made initial attempts to screen and identify compounds capable of disrupting TCF/β-catenin complexes 83. They screened chemical libraries of small molecules using a high-throughput assay system and found two potent inhibitors (PKF115-584 and PKF222-815) capable of disrupting TCF/β-catenin complexes and antagonize cellular effects of β-catenin-dependent activities 83. They also identified other β-catenin inhibitors (PKF118-310, CGP049090 and PKF118-744) capable of inhibiting β-catenin activity 83. Furthermore, Wnt/β-catenin signalling inhibitor PKF118–310 effectively inhibited proliferation of prostate cancer cells (IC50 ≤ 1 μM) 84. Minke *et al*. and Gandhirajan *et al*. investigated the effects of CGP049090 (IC50 ≤ 1 μM) and PFK115-584 (IC50 ≤ 1 μM) in acute myeloid leukaemia (AML) and chronic lymphocytic leukaemia (CLL) cells respectively 85, 86.They found that both compounds led to a substantial decrease in the expression of β-catenin/LEF1 target genes (*e.g*. c-myc, cyclin D1 and survivin). Down-regulation of these survival-related genes resulted in the induction of cell death in AML cell lines and cells derived from AML patients 86. These inhibitors also induced cell death in CLL cell lines and patient samples 87. PKF118-310 was also found to be effective against human osteosarcoma cells. Here, inhibition of β-catenin resulted in suppression of MMP9 enzymatic activity and thus reduced cancer cell invasion and migration 87. Apart from its anti-invasive effects, PKF118-310 also induced cell death and G2/M phase arrest in osteosarcoma cells by decreasing expression of cyclin D1, c-Myc and survivin 87. Hallett *et al*. found that PKF118-310 (IC50 ≤ 1 μM) was effective against breast cancer initiating cells (BTIC) where it inhibited tumour growth and proliferation 88. Administration of PKF118-310 to tumour-bearing mice halted tumour growth *in vivo* and viable tumour cells harvested from PKF118-310 treated mice were unable to induce the growth of secondary tumours after transplantation 88. Emami *et al*. identified a novel inhibitor (ICG-001; IC50 ≤ 3 μM) of beta-catenin/CREB-binding protein transcription activity. ICG-001 induced apoptosis in transformed cells selectively and also reduced *in vitro* and *in vivo* growth of colon carcinoma cells 89. In another attempt to identify novel inhibitors of the Wnt/β-catenin pathway, Ewan *et al*. screened a chemical library against a transcription factor reporter cell line in which the activity of the pathway was induced at the level of dishevelled (dsh) protein 90. They identified a potent inhibitor CCT036477 (IC50 ≤ 5 μM), capable of inhibiting TCF/β-catenin-mediated transcription and inducing cancer cell death 90. Chen *et al*. identified nine potent β-catenin inhibitors (IC50 ≤ 2.5 μM) 91. They screened over 200 thousand compounds *in vitro* to identify less toxic and highly selective inhibitors against the Wnt/β-catenin signalling pathway 91. Based on the results using cellular systems, five compounds were found to inhibit Wnt response (IWR) and four compounds were found to inhibit Wnt production (IWP) 91. Huang *el al*. identified a novel inhibitor (XAV939) which antagonized Wnt/β-catenin pathway by inhibiting tankyrase 92. Tankyrase is an axin inhibitor, thus XAV939 increases axin levels in cells 92. Axin stabilization further leads to β-catenin degradation and Wnt/β-catenin pathway inhibition 92. Song *et al*. employed a high-throughput screen to identify inhibitors of Wnt/β-catenin signalling 93. They found a special class of compounds (acyl hydrazones; IC50 ≤ 2 μM) with iron chelating activity 93. They demonstrated that their inhibitory effect on the Wnt/β-catenin signalling pathway is linked to iron chelation 93. These results further supported the initial finding of Brookes *et al*. that iron can induce Wnt/β-catenin signalling 93, 94. Recently, Coombs *et al*. used a cell-based assay system as well as transgenic MMTV-Wnt1 and MMTV-PyMT mice models to screen Wnt/β-catenin inhibitors 95. They found a compound N-((8-hydroxy-7-quinolinyl) (4-methylphenyl)methyl)benzamide (HQBA) with IC50 ranging between <1 nM and 50 μM in various cellular models 95. In mice models, it effectively reduced tumour mass 95. HQBA was found to be safe at higher doses (60–90 mg/kg) and interestingly its anticancer effects were also caused by iron chelation 95. In various other attempts to identify β-catenin inhibitors, many potent compounds capable of inhibiting β-catenin activity as well as its molecular interactions were identified. Some of these inhibitors are listed in Table 2.

**Table 2:** Small molecular inhibitors of β-catenin signaling

S.no.	Inhibitor	Target	Reference
1	PKF118-310, CGP049090, PKF115-584, PKF222-815 and PKF118-744	β-catenin–TCF interaction	83
2	ICG001	β-catenin–CBP interaction	89
3	CCT036477	β-catenin–TCF interaction	90
4	XAV939	Tankyrase	92
5	Acyl hydrazones, HQBA	Iron chelators	93, 95
6	Molecules with 2,3,6-trisubstituted pyrido[2,3,-b] pyrazine core skeletons	β-catenin	119
7	Carnosic acid	β-catenin/BCL9	120
8	CCT031374	β-catenin	121
9	iCRT-3,5,14, NC043	β-catenin–TCF interaction	122, 123
10	Ibuprofin, aspirin	Cox2 Inhibitors	124

Furthermore, inhibition of β-catenin can also be employed against cancer stem cells and chemo-resistant cancer cells. The Rosen laboratory evaluated radiation resistance in CSCs isolated from p53-null mouse mammary tumours 96. Using the inhibitor perifosine, they were able to block AKT and β-catenin activation and sensitize the cells to radiation 81. Another study has shown that β-catenin is a target of selenium and its inhibition is associated with increased chemosensitivity to cytotoxic drugs in various human cancers 96. However, there are only limited reports detailing the toxicological, pharmacokinetic and pharmacodynamic data for these inhibitors. Collectively, the studies carried out using small molecule inhibitors of β-catenin targeted to inhibit cancer progression look promising. These small molecule inhibitors reduce cancer growth, induce apoptosis, decrease invasion and migration of cancer cells.

## Plant-derived beta-catenin modulators

Various plant-derived compounds with anticancer activities are also known to inhibit or modulate the Wnt/β-catenin signalling pathway. Tetrandrine (TET), a bis-benzylisoquinoline alkaloid purified from the root of *Stephania tetrandra* exhibited significant anticancer activity by inhibiting β-catenin/Tcf transcriptional activity (IC50 range, 1.25–5.7 μM) 97. Curcumin, a plant-derived natural phenol from the popular Indian spice turmeric shows excellent tumour inhibition property without significant toxicity 98. Curcumin and its derivative CHC007100 inhibit β-catenin/Tcf signalling by 58–63% and 70–78%, at 20 and 100 μM doses respectively 98. Another plant-derived flavonoid quercetin also leads to the decrease in beta-catenin/Tcf transcriptional activity 99. Quercetin acts at a very high dose (IC50, 100 μM) and further investigation is required for data related to its safety and efficacy. Plant flavanoid silymarin, from *Silybum marianum*, inhibits melanoma cell migration (IC50 ≤ 20 μM) by inhibiting β-catenin nuclear localization 100. Carnosol, from the herb rosemary, prevents APC-associated intestinal tumourigenesis in a mouse model of colonic tumourigenesis. Its dietary administration (0.1%) reduced tumour growth by 46 per cent without any toxicity. It suppressed tumour growth *via* its ability to enhance E-cadherin-mediated adhesion and inhibition of β-catenin tyrosine phosphorylation 101. Cardamonin a natural compound derived from *Aplinia katsumadai* inhibits 65–70 per cent of β-catenin activity at a dose ≤10 μM, without compromising cell viability 102. These studies indicate that various plant-derived chemicals (phytochemicals) and their various analogues can also modulate β-catenin functions and thus could be tested against various cancers with abnormal β-catenin activity. These phytochemicals and their derivatives further require thorough investigation for their safety and efficacy.

## Conclusions and perspectives

The potential of the pharmacological modulation of β-catenin in cancer therapeutics is paramount. This may possibly provide an attractive option of targeting various aspects of the carcinogenic process *i.e*. initiation, progression and chemoresistance in conjunction with the traditional chemotherapy. However, the long-term effects of the pharmacological manipulation of β-catenin remain still unclear. The overall regulation of β-catenin involves multiple signalling pathways and therefore pharmacological modulation could be counterbalanced through the activation of compensatory signalling pathways. The possibility of adverse side effects of β-catenin inhibition cannot be ruled out at this juncture and more detailed studies will be required to address this key issue. To date, use of various small molecule inhibitors of β-catenin targeting cancer have provided some encouraging results. Further efforts can be directed towards evaluating the efficacy of the existing inhibitors in variety of cancer types, stages and especially against cancer initiating cells/cancer stem cells and chemo-resistant cancers. As it is evident that microenvironmental regulation of the β-catenin activity plays a central role in the malignant transformation and induction of metastasis; these inhibitors can also be used in combination with inhibitors of cancer survival pathways and modulators of tumour microenvironment. Some of the phytochemicals that seem to modulate β-catenin activity can also be used as lead compounds for developing β-catenin-targeted therapeutics. Targeting Wnt–β-catenin activity could open new avenues for novel and tailor-made cancer therapeutic approaches.
